# Inflammatory Biochemical Mediators and Their Role in Myofascial Pain and Osteopathic Manipulative Treatment: A Literature Review

**DOI:** 10.7759/cureus.22252

**Published:** 2022-02-15

**Authors:** Benjamin T Leicht, Christina Kennedy, Chante Richardson

**Affiliations:** 1 Department of Research, Alabama College of Osteopathic Medicine, Dothan, USA; 2 Physiology, Alabama College of Osteopathic Medicine, Dothan, USA; 3 Biochemistry, Alabama College of Osteopathic Medicine, Dothan, USA

**Keywords:** myofascial pain, inflammation, il-1β, tnf-α, omt

## Abstract

Chronic inflammatory pain conditions, specifically myofascial pain (MFP), account for an overwhelming percentage of office visits every day. The combination of the high cost of its treatment and frequent patient visits makes MFP a critical pathology to be investigated. Sharpening our understanding of the molecular mechanisms within MFP will expedite the enhancement of therapeutic approaches. Inflammation plays a critical role in the pathophysiology of MFP. The chief inflammatory mediators of interest in this review related to MFP are interleukin 1β (IL-1β), IL-6, and tumor necrosis factor-α (TNF-α). This review aimed to determine the impact of inflammatory mediators on fibroblasts and satellite cells, specifically their role in muscle injury and regeneration. Blocking pro-inflammatory mediators such as IL-1β, IL-6, and TNF-α in these cell types could prove to be an effective treatment for MFP. An osteopathic manipulative treatment (OMT) modality, specifically indirect counterstrain therapy, was investigated in the hopes of elucidating a reduction in particular cytokines. In addition, myofascial release (MFR) therapies (OMT modality) were explored as a potentially effective treatment through the acceleration of wound healing, stimulation of muscle regeneration, and decreased inflammation via altered fibroblast activity. Pharmacologic agents such as non-steroidal anti-inflammatory drugs (NSAIDs) are commonly used to treat MFP but have a higher adverse side effect profile compared to OMT therapy. The optimal management of MFP is likely multifactorial, and more treatment modalities must be explored.

This literature review analyzed 17 peer-reviewed articles specifically related to MFP management and the role of inflammation in MFP. Chronic inflammation from other etiologies was excluded. Our aim was to elucidate the biochemical mechanisms underlying MFP and inflammation in an effort to promote the medical community's understanding of treatment modalities for this chronic condition. This study revealed that various OMT techniques such as MFR and counterstrain lead to changes on the cellular level in MFP. Discovering similar effects on biochemical inflammatory markers with non-pharmacologic treatment modalities was an exciting revelation and one that could potentially change the way physicians address pain management.

## Introduction and background

Discussing myofascial pain (MFP) is difficult without first defining myofascial tissue. The myofascial tissue is a continuous connective tissue layer throughout the body that provides stabilization and connection to muscles, anchoring them to the overlying skin. This layer of connective tissue is composed of a dense network of collagen, providing structural support and elastin, for elastic function. The primary cell type in the myofascia are fibroblasts, which play a major role in the tissue injury and repair process. Adipocytes and a wide array of leukocytes can also be identified within the myofascial tissue [1].

MFP is defined as a musculoskeletal condition characterized by palpable myofascial trigger points. A trigger point is a tight band of muscles that is tender upon palpation, eliciting pain. Additional diagnostic tools such as electromyography, MRI, and ultrasound have been shown to be useful in elucidating trigger points in the musculature as well [2]. These trigger points are often utilized as the diagnostic criteria for this condition, and there is a wide array of treatment modalities to manage it. Other findings include autonomic alterations such as diaphoresis, erythema, redness, and swelling [2]. An analysis of the pathophysiology of MFP usually begins with the prevailing theory of dual innervation at the neuromuscular endplate [3]. One particular study gathered the prevailing data on innervation at the neuromuscular endplate, which has dominated the field since the 1930s. This data has suggested that the neuromuscular endplate is innervated with both somatic and sympathetic nerve fibers [3]. When dysfunction occurs in this dual innervation model, this irregular activity propagates the release of various inflammatory mediators that stimulate the nociceptive pathway. In addition, satellite cells and fibroblasts become activated in an effort to initiate and execute muscle injury repair [4-6]. Satellite cells function as the quiescent and active stem cells of muscle tissue, activated during muscle injury for the regeneration of myofibers [7].

Inflammation plays a critical role in the pathophysiology of MFP. The key inflammatory mediators involved in MFP are interleukin-1β (IL-1β), IL-6, IL-8, tumor necrosis factor-α (TNF-α), substance P, bradykinin, norepinephrine, calcitonin gene-related peptide, and serotonin [8]. This study will focus predominately on the cytokines: IL-1β and TNF-α. TNF-α is considered a pro-inflammatory cytokine that becomes stimulated by a plethora of cells including but not limited to monocytes, fibroblasts, adipocytes, and smooth muscle cells [9]. The mechanism of action for TNF-α stimulation involves a large cascade of events that can be simplified into the following major steps: TNF-α binding to TNF receptor 1 (TNFR1), which communicates with TNF receptor-associated death domain (TRADD), which then associates with TNF receptor-associated factor 2 (TRAF2) and receptor-interacting protein to ultimately activate nuclear factor kappa-light-chain-enhancer of activated B cells (NF-κB) [10]. IL-1β has been extensively studied and demonstrated to enhance various fibroblastic functions including those assisting with repair and inflammation [11]. The functions of these mediators are summarized in Table 1 below. In addition to an array of biochemical mediators, inflammation in MFP involves the following cells: fibroblasts, satellite cells, and macrophages [5-6]. Fibroblasts play a role in the regeneration of muscle tissue damaged by trigger points. Fibroblasts work to additionally organize and synthesize collagen in the fascia, while also stimulating pro-inflammatory cytokines (TNF-α, IL-1β). Macrophages and granulocytes are among the primary cells involved in activating and propagating the inflammatory response. The anti-inflammatory macrophage phenotype is thought to be linked with the initiation of myogenic differentiation in muscle injury repair involving satellite cells, while myotubule reconstruction via fibroblasts occurs further downstream in the repair process [5-6]. The effects of osteopathic manipulation treatment (OMT) and various other treatment modalities are investigated in an effort to unveil the biochemical and cellular changes involved in the currently applied therapeutic methods for patients with MFP.

This literature review was previously presented virtually as a poster at the Society of Hospital Medicine's Annual Wiregrass Chapter Meeting and Poster Competition event on November 7, 2020.

## Review

Methods

In this literature review, papers focusing on MFP as the etiology behind inflammatory reactions were chosen while papers addressing inflammatory reactions from other etiologies were excluded. A total of 17 articles were selected and reviewed extensively to unveil the underlying molecular mechanisms linking MFP to inflammatory reactions. These molecular pathways were then utilized to discover articles discussing the treatment modalities for MFP in an effort to further understand the molecular mechanisms underlying MFP treatment via OMT.

Discussion

Biochemical Mediators and Cells in MFP and the Inflammatory Pathway

MFP shares several pro-inflammatory mediators with the inflammatory pathway. Common pro-inflammatory mediators include vasoactive amines (histamine), eicosanoids, cytokines (IL-1β, IL-6, IL-8, IL-12, IL-17, and TNF-α), acute-phase proteins, and monocytes [12]. Those of particular interest in MFP include TNF-α, bradykinin, IL-1β, IL-6, IL-8, and substance P, as highlighted in Table 1 [8]. TNF-α has a unique role in these pathways, as it has been shown to be active in fibroblasts and smooth muscle cells of damaged muscle tissue. When released, TNF-α binds the TNFR1 receptor, stimulating a pro-inflammatory cascade involving TRADD, TRAF2, and receptor-interacting protein. The flow of this cascade ultimately actives NF-kB, further enabling the propagation of the inflammatory response [10]. β-endorphin is an additional relevant biochemical mediator in the MFP and inflammatory pathways. This neuropeptide was demonstrated to reduce levels of substance P, facilitating an analgesic effect [13]. β-endorphin’s pain suppression mechanism has made it an excellent analyte to quantify for various MFP treatments. The specific techniques and corresponding β-endorphin responses are discussed in the MFP management section below.

**Figure 1:**
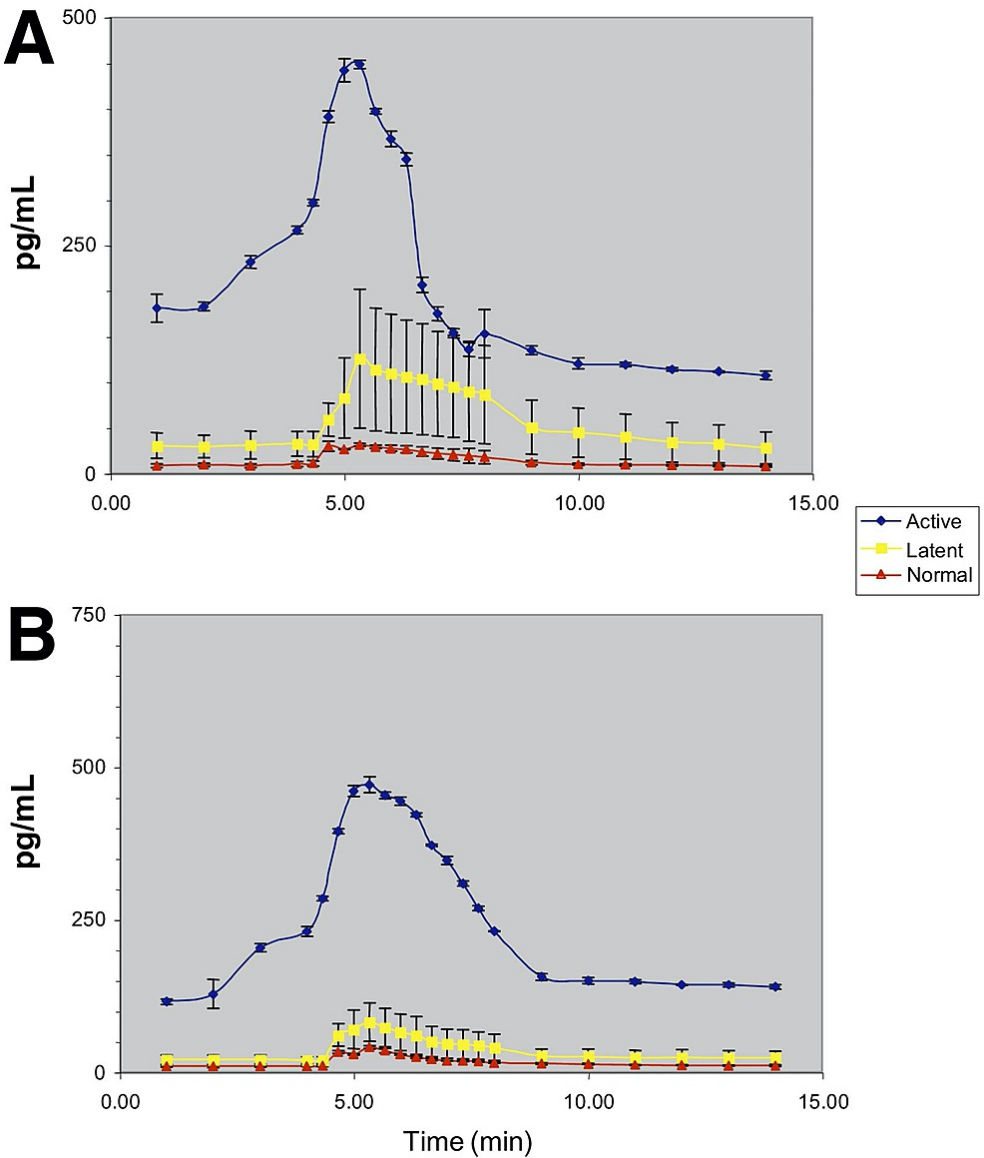
Analyte concentrations in the trapezius for (A) TNF-α and (B) IL-1β* *[8]. Permission to use figures obtained from original author and publisher IL: interleukin; TNF: tumor necrosis factor

**Figure 2:**
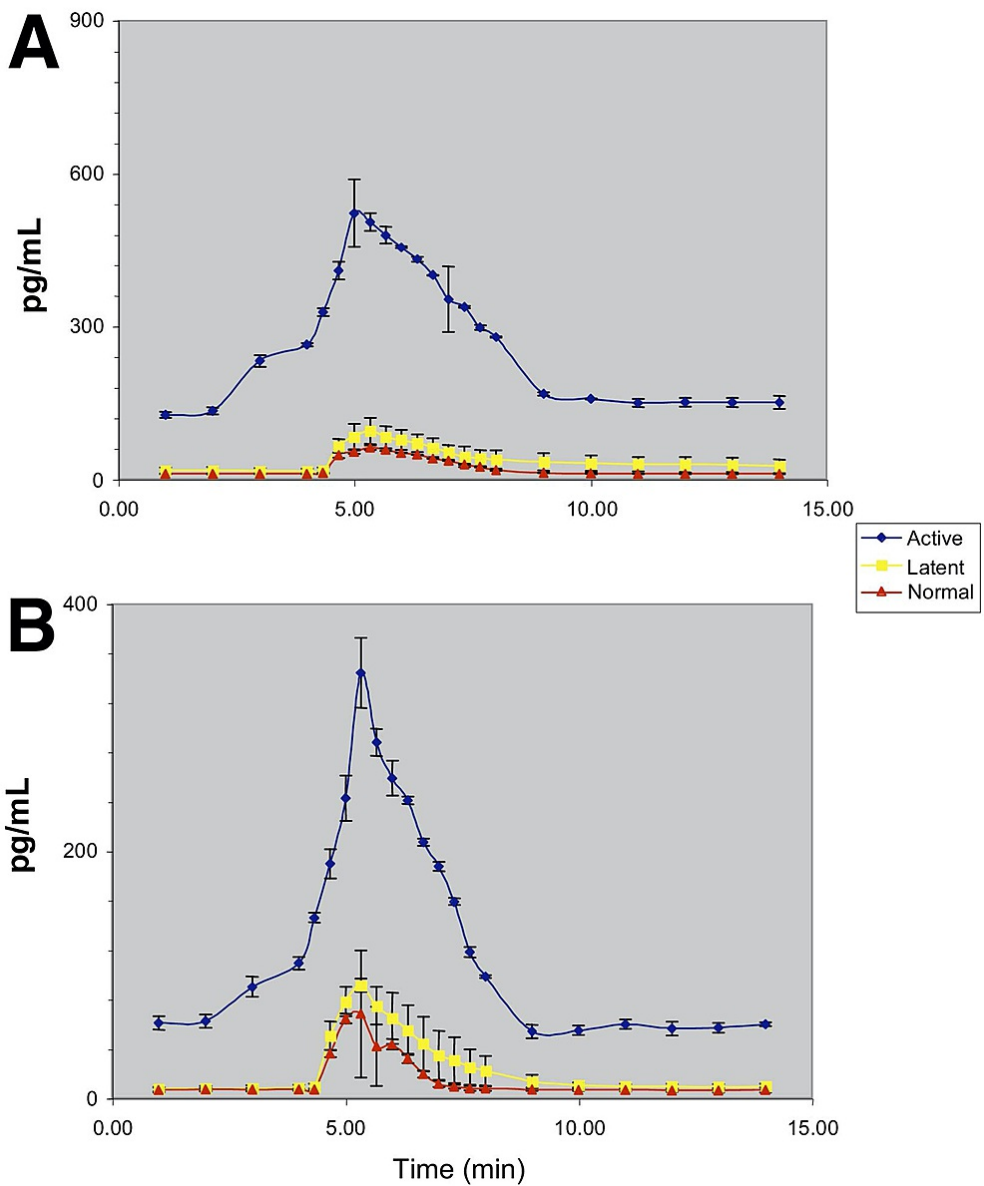
Analyte concentrations in the trapezius for (A) IL-6 and (B) IL-8* *[8]. Permission to use figures obtained from original author and publisher IL: interleukin

Understanding the role of fibroblasts, satellite cells, and other myogenic precursor cells during muscle injury is critical to discerning the underlying biochemical mechanism of many therapeutic options for musculoskeletal-related pain syndromes such as MFP. Fibroblasts work in conjunction with myogenic precursors to synthesize, repair, and organize collagen during tissue injury. Pro-inflammatory mediator release during muscle injury is linked to fibroblast stimulation, further asserting the active role fibroblasts play in the pro-inflammatory pathway [6]. Satellite cells, stimulated by transforming growth factor-beta (TGF-β), have proven to be equally involved in the tissue repair pathway, facilitating tissue regeneration and myotubule reconstruction. They function as quiescent stem cells, activated when muscle repair is required [7]. TGF-β acts primarily as an anti-inflammatory mediator promoting extracellular matrix reconstruction through collagen deposition via fibroblast activation [14]. The activity of this growth factor has been proven to play an integral role in the muscle regeneration process through its continuous interactions with fibroblasts and satellite cells. Other cells of importance to the inflammatory pathway include macrophages and granulocytes. Both cell types are responsible for the release of various pro-inflammatory cytokines such as IL-1, IL-6, and IL-8 as seen in Figure 1 and Figure 2. However, neutrophils, mast cells, and macrophages are additionally known for stimulating the release of IL-17, which is the predominant pro-inflammatory chemotactic factor for cells responding to tissue damage [15]. Table 1 illustrates the pro-inflammatory mediators and their functions.

**Table 1:** Pro-inflammatory mediators and their functions IL: interleukin; TNF: tumor necrosis factor

Chemical mediator	Function
Bradykinin	Pro-inflammatory peptide; stimulates pain pathway and prostaglandin synthesis
TNF-α	Pro-inflammatory cytokine; stimulates NE and acute-phase protein production; also induces nociception
Substance P	Neuropeptide involved in the nociceptive pathway; stimulates mast cell degranulation, histamine, and serotonin release
IL-1β	Pro-inflammatory cytokine; stimulates acute-phase protein production and can induce nociception
IL-6	Pro/anti-inflammatory cytokine; stimulates acute-phase protein production and can induce nociception
IL-8	Pro-inflammatory cytokine; stimulates acute-phase protein production and can induce nociception
Serotonin (5-HT)	Involved in pain pathway and prostaglandin synthesis, and vasoconstrictive effects during injury
Norepinephrine (NE)	Increases blood pressure via vasoconstriction
Calcitonin gene-related peptide (CGRP)	Neuropeptide involved in the nociceptive pathway

Biochemical Markers Linking MFP With the Inflammatory Pathway

Several studies have been conducted to explore which biochemical mediators are active in individuals with MFP and how the concentrations of those analytes compare to those in patients without MFP. One such study confirmed a decreased pH and increased bradykinin, substance P, calcitonin gene-related peptide, TNF-α, IL-1β, IL-6, IL-8, serotonin, and norepinephrine in those with MFP [8]. Table 1 highlights each of these pro-inflammatory mediators that were observed in this study to have lower concentrations in individuals without MFP. Figure 1 and Figure 2 elucidate the rise in TNF-α, IL-1β, IL-6, and IL-8 during the inflammatory response to muscle injury. Understanding the levels of these markers during chronic inflammatory states will aid in providing the most effective treatment options for patients. The same study mentioned above confirmed additional elevated levels of pro-inflammatory markers at sites unrelated to the active myofascial trigger points, confirming a systemic manifestation of elevated inflammatory markers [8].

Management of MFP

The treatment modalities for MFP are vast, consisting of a large array of pharmacologic and non-pharmacologic options. In a majority of cases, a combination of various treatments often serves as most efficacious to the patient in terms of reducing pain associated with myofascial trigger points. Common pharmacologic avenues consist of NSAIDs, benzodiazepines, muscle relaxers, tramadol, selective serotonin reuptake inhibitors (SSRIs), serotonin-norepinephrine reuptake inhibitors (SNRIs), and tricyclic antidepressants (TCAs) [16]. NSAIDs have been shown to have little efficacy in the treatment of MFP but are still commonly used due to their easy availability. Some topical NSAIDs have proven effective but are often expensive [16]. Some muscle relaxers have shown efficacy in treating MFP; however, they are often accompanied by unwanted side effects (sedation, hypotension). TCAs such as amitriptyline have been used but they lead to considerable side effects, while further studies are required in the case of SNRIs and SSRIs to determine their efficacy [16]. Many non-pharmacologic interventions are utilized as well to alleviate pain in MFP. OMT is one of the predominant non-pharmacologic methods employed by many. The mechanisms and techniques of OMT will be discussed further in the section below on the molecular outcomes of OMT. Other interventions include exercise, postural corrections, stress reduction, acupuncture, needling, and laser therapy, all of which have demonstrated effective alleviation of symptoms [13,16-17]. Choosing OMT over other pharmacologic interventions provides effective treatment for the patient without the harmful side effects that many of the drugs cause.

Overall, the treatment for MFP is multifactorial; the risks and rewards of each option should be considered, and more research is required to understand the efficacy of these treatments [16].

Cellular and Biochemical Effects of Non-pharmacologic MFP Treatment on the Inflammatory Pathway

**Figure 3:**
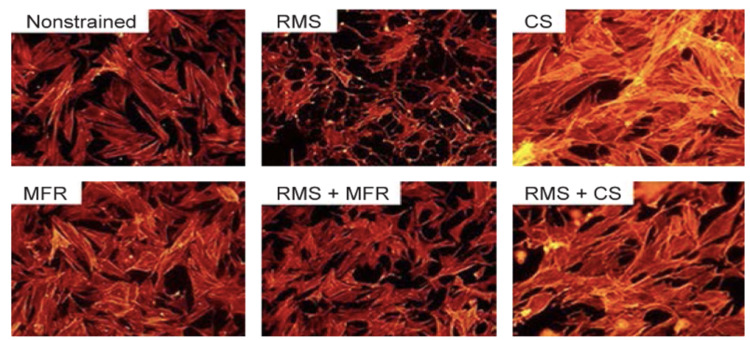
The effect of different osteopathic manipulative treatment modalities on fibroblast morphology and physiology* *[6]. Permission to use figures obtained from original author and publisher RMS: repetitive motion strain; CS: counterstrain; MFR: myofascial release

Understanding the molecular mechanisms of non-pharmacologic interventions such as OMT, stretching, exercise, laser therapy, and needling is essential for the refinement of the current techniques and the development of novel methods. One study examined the effects of static stretching, counterstain, and myofascial release (MFR) techniques [6]. Static stretching was demonstrated to increase the cross-sectional area of fibroblast bodies, correlating with a localized decrease in tissue tension. MFR was proven to enhance wound healing, stimulate muscle regeneration, and decrease inflammation by affecting fibroblast activity. As seen in Figure 3, the regeneration of damaged muscle tissue is visualized in vitro after treatment with MFR. The exact mechanism by which this occurs was not elucidated. Indirect techniques such as counterstrain were shown to reduce levels of IL-6 [9]. One study demonstrated a measurable decrease in the levels of substance P and a significant increase in the levels of β-endorphin (endogenous opioid) in individuals with myofascial trigger points treated with dry needling, advocating dry needling as an effective MFP therapy [13]. A similar study examined the molecular effects of low-level laser (LLL) therapy on active myofascial trigger points in rabbits. Levels of TNF-α and substance P were significantly lower in groups that received LLL treatment, demonstrating its efficacy as an intervention for MFP [15]. Specific OMT techniques such as MFR have also shown efficacy in practice, demonstrated through patient pain relief. However, concentrations of various inflammatory analytes during OMT treatment have yet to be revealed. The literature on this topic is sparse and demonstrates a need for future research in order to enhance the medical community's understanding of how OMT works on the molecular level, specifically with the inflammatory pathway and fibroblasts. However, some studies have been conducted to analyze the effects of OMT on fibroblasts. For example, MFR has been shown to facilitate fibroblast-mediated myoblast differentiation and improve impaired repetitive muscle strain wound healing [6].

## Conclusions

Specific biochemical mediators involved in the inflammatory pathway have been demonstrated to have a role in MFP and its treatment. The activity of these various markers is influenced by multiple non-pharmacologic OMT treatments including but not limited to MFR and counterstrain. Given this data, future research should be pursued in an effort to further unveil the therapeutic benefits of OMT on a cellular level. Once a better understanding of these mechanisms is gained, a potential decline in the overuse of pharmacologic intervention could be achieved.
